# Supplementary material for: Bacteroides is increased in an autism cohort and induces autism-relevant behavioral changes in mice in a sex-dependent manner
Source: NPJ Biofilms Microbiomes. 2023 Dec 18;9:103. doi: 10.1038/s41522-023-00469-2 (PMC10728123; doi:10.1038/s41522-023-00469-2)
Supplement: Supplementary file 1 — Reporting summary [file 41522_2023_469_MOESM1_ESM.pdf]

Corresponding author(s): Evan Elliott

Last updated by author(s): Oct 1, 2023

Reporting Summary

Nature Portfolio wishes to improve the reproducibility of the work that we publish. This form provides structure for consistency and transparency in reporting. For further information on Nature Portfolio policies, see our Editorial Policies and the Editorial Policy Checklist.

Statistics

For all statistical analyses, confirm that the following items are present in the figure legend, table legend, main text, or Methods section.

n/a	Confirmed	
☐	☒	The exact sample size (n) for each experimental group/condition, given as a discrete number and unit of measurement
☐	☒	A statement on whether measurements were taken from distinct samples or whether the same sample was measured repeatedly
☐	☒	The statistical test(s) used AND whether they are one- or two-sided<br>*Only common tests should be described solely by name; describe more complex techniques in the Methods section.*
☐	☒	A description of all covariates tested
☐	☒	A description of any assumptions or corrections, such as tests of normality and adjustment for multiple comparisons
☐	☒	A full description of the statistical parameters including central tendency (e.g. means) or other basic estimates (e.g. regression coefficient) AND variation (e.g. standard deviation) or associated estimates of uncertainty (e.g. confidence intervals)
☐	☒	For null hypothesis testing, the test statistic (e.g. F, t, r) with confidence intervals, effect sizes, degrees of freedom and P value noted<br>*Give P values as exact values whenever suitable.*
☒	☐	For Bayesian analysis, information on the choice of priors and Markov chain Monte Carlo settings
☒	☐	For hierarchical and complex designs, identification of the appropriate level for tests and full reporting of outcomes
☒	☐	Estimates of effect sizes (e.g. Cohen's d, Pearson's r), indicating how they were calculated

Our web collection on statistics for biologists contains articles on many of the points above.

Software and code

Policy information about availability of computer code

Data collection	No software was used
Data analysis	No software was used

For manuscripts utilizing custom algorithms or software that are central to the research but not yet described in published literature, software must be made available to editors and reviewers. We strongly encourage code deposition in a community repository (e.g. GitHub). See the Nature Portfolio guidelines for submitting code & software for further information.

Data

Policy information about availability of data

All manuscripts must include a data availability statement. This statement should provide the following information, where applicable:

- Accession codes, unique identifiers, or web links for publicly available datasets
- A description of any restrictions on data availability
- For clinical datasets or third party data, please ensure that the statement adheres to our policy

All data, including sequencing data and metadata, used in this manuscript will be freely available to the research community. 16S rRNA sequencing data of human samples are provided at the gene expression omnibus at GSE244391.

Research involving human participants, their data, or biological material

Policy information about studies with human participants or human data. See also policy information about sex, gender (identity/presentation), and sexual orientation and race, ethnicity and racism.

Reporting on sex and gender	We reported on sex in the main results section. This is what was reported: Sex ratio: Control group 30 males 12 females, Autism group 72 males 25 females. fisher's exact test p=0.83". Sex was used as a covariate in the study.
Reporting on race, ethnicity, or other socially relevant groupings	There was no race or ethnicity data collected or used in this study. All study participants are residents of the country of Israel.
Population characteristics	The covariate population characteristics that were used were age, sex, and diet (gluten free, dairy free, and vitamin supplementation).
Recruitment	Patients were recruited through the Autism Registry and Biobank. This is a large recruitment process for several autism studies that is based mainly in northen Israel.
Ethics oversight	Helsinki committee of Ziv medical center (Safed, ISrael)

Note that full information on the approval of the study protocol must also be provided in the manuscript.

Field-specific reporting

Please select the one below that is the best fit for your research. If you are not sure, read the appropriate sections before making your selection.

☒ Life sciences ☐ Behavioural & social sciences ☐ Ecological, evolutionary & environmental sciences

For a reference copy of the document with all sections, see nature.com/documents/nr-reporting-summary-flat.pdf

Life sciences study design

All studies must disclose on these points even when the disclosure is negative.

Sample size	For mouse studies, we aimed to perform behavioral experimentation on 12 animals per group, which is the gold standard for behavioral animal epxerimentation.
Data exclusions	No data was excluded.
Replication	All data was replicated as described in the manuscript
Randomization	All animals were randomly and blindly split to experimental groups. In the case of human samples, they were split to autism and control, which is not a random assignment, but based on diagnosis.
Blinding	All experimenters were blinded to the sample or animal indentity at the time of experimentation.

Reporting for specific materials, systems and methods

We require information from authors about some types of materials, experimental systems and methods used in many studies. Here, indicate whether each material, system or method listed is relevant to your study. If you are not sure if a list item applies to your research, read the appropriate section before selecting a response.

Materials & experimental systems

n/a	Involved in the study	
☒	☐	Antibodies
☒	☐	Eukaryotic cell lines
☒	☐	Palaeontology and archaeology
☐	☒	Animals and other organisms
☒	☐	Clinical data
☒	☐	Dual use research of concern
☒	☐	Plants

Methods

n/a	Involved in the study	
☒	☐	ChIP-seq
☒	☐	Flow cytometry
☒	☐	MRI-based neuroimaging

Animals and other research organisms

Policy information about studies involving animals; ARRIVE guidelines recommended for reporting animal research, and Sex and Gender in Research

Laboratory animals	C57/black mice were used in this study.
Wild animals	This study did not involve wild animals.
Reporting on sex	We reported on sex throughout the manuscript. As described in the manuscript, our findings in animal models were sex-specific.
Field-collected samples	Our study did not involve samples from the field.
Ethics oversight	All procedures were approved by the Animal Ethics Committee of the Bar-Ilan University, Israel (protocol number 36-06-2019).

Note that full information on the approval of the study protocol must also be provided in the manuscript.
